# How Active Are Porcine Endogenous Retroviruses (PERVs)?

**DOI:** 10.3390/v8080215

**Published:** 2016-08-03

**Authors:** Joachim Denner

**Affiliations:** Robert Koch Institute, Nordufer 20, 13353 Berlin, Germany; DennerJ@rki.de; Tel.: +49-30-187542800

**Keywords:** porcine endogenous retroviruses, human endogenous retroviruses, xenotransplantation, retroviruses

## Abstract

Porcine endogenous retroviruses (PERVs) represent a risk factor if porcine cells, tissues, or organs were to be transplanted into human recipients to alleviate the shortage of human transplants; a procedure called xenotransplantation. In contrast to human endogenous retroviruses (HERVs), which are mostly defective and not replication-competent, PERVs are released from normal pig cells and are infectious. PERV-A and PERV-B are polytropic viruses infecting cells of several species, among them humans; whereas PERV-C is an ecotropic virus infecting only pig cells. Virus infection was shown in co-culture experiments, but also in vivo, in the pig, leading to de novo integration of proviruses in certain organs. This was shown by measurement of the copy number per cell, finding different numbers in different organs. In addition, recombinations between PERV-A and PERV-C were observed and the recombinant PERV-A/C were found to be integrated in cells of different organs, but not in the germ line of the animals. Here, the evidence for such in vivo activities of PERVs, including expression as mRNA, protein and virus particles, de novo infection and recombination, will be summarised. These activities make screening of pigs for provirus number and PERV expression level difficult, especially when only blood or ear biopsies are available for analysis. Highly sensitive methods to measure the copy number and the expression level will be required when selecting pigs with low copy number and low expression of PERV as well as when inactivating PERVs using the clustered regularly interspaced short palindromic repeats (CRISPR)/CRISPR-associated nuclease (CRISPR/Cas) technology.

## 1. Introduction

Endogenous retroviruses are the result of an infection of germ cells with a retrovirus leading to the integration of the viral genome as DNA copy (provirus) in all cells of the organism [1,2,3]. Human endogenous retroviruses (HERVs), especially HERV-K, are well characterised and it has been shown that most of them are defective: if they are expressed in human tumour cells, they are rarely released as particles and even if they are not defective then they are not infectious [4]. Since the integration of HERVs took place millions of years ago and HERVs are inactive, the copy number per cell is identical in all cells of a human individual. Concerning (i) the integration site; (ii) the presence of all proviruses and (iii) mutations in the viral sequences, minor differences exist between the individuals. Although HERVs are replication inactive, it is important to note that proteins encoded by HERVs and endogenous retroviruses of other mammalians are utilised for physiological functions of the host. The Env proteins are required for the generation of a functional placenta using the fusion competence of this protein (for review see [5]). Interestingly, in different species different proviruses were utilised for this function. In addition, Env proteins may be involved in immunosuppression required for the survival of the embryo [6] and recent data suggest that endogenous retroviruses are involved in the regulation of the innate immunity [7]. In contrast to HERVs, endogenous retroviruses of many other species, especially endogenous gammaretroviruses, are released as virus particles and can infect cells of their own species and/or of other species [8].

The porcine endogenous retroviruses (PERVs) are not very well studied. PERVs are gammaretroviruses closely related to murine leukaemia viruses (MuLV), feline leukaemia viruses (FeLV) and koala retroviruses (KoRV). PERVs are of interest in the context of xenotransplantation, which is under development in order to alleviate the shortage of human tissues and organs for the treatment of tissue and organ failure. Pigs are for several reasons (e.g., size, physiological similarity with humans, availability, multiparity, availability of multiple genetic changes to prevent immunological rejection) the preferred donor animals. PERVs are released from normal pig cells and are infectious. PERV-A and PERV-B are polytropic viruses infecting cells from several species among them humans, and therefore they pose a risk for xenotransplantation. PERV-C is an ecotropic virus infecting only pig cells. Virus infectivity was shown in co-culture experiments in vitro (for review see [9,10]). In recent years, evidence accumulated that PERVs are—in contrast to HERVs—still active in vivo, in the pig. In contrast to HERVs which are expressed as mRNA, protein and non-infectious particles, PERVs are still more active in vivo, in the pig. Here, data demonstrating the activity of PERVs, e.g., the expression of the proviruses, their replication, de novo integration in vivo, and recombination in pigs will be analysed, and the implication for testing pigs for xenotransplantation will be discussed.

## 2. Evidence for Replication and de Novo Integration in Vivo

In the case of HERVs, the copy number per cell is identical in all cells of one individual and is more or less identical in all individuals of the species. This is true for example for HERV-K which was introduced into the human germ line 28 millions years ago [11]. Findings that PERV-A and PERV-B are present in *Suiformes*, and PERV-C was detectable only in *Sus scrofa* and in closely related species, suggested an African origin of PERV of about 7.5 million years ago. It seems likely that PERV-C originated more recently (1.5 to 3.5 million years ago) by recombination with unknown homologous sequences [12,13].

The copy numbers of PERV in different breeds, in different animals of one breed, in different organs of one and the same animal and in pig cell lines are poorly studied (Table 1) [14,15,16,17,18,19,20,21,22,23,24,25,26]. In Chinese miniature pigs the copy number in the genomic DNA was reported to vary significantly, ranging from four to 96 copies [17]. Some animals had a low number of proviruses, a low expression at the RNA level and were PERV-C free. The absence of PERV-C prevents the formation of recombinant PERV-A/C variants which can replicate at a higher rate when compared with the maternal PERV-A and which can infect in contrast to PERV-C human cells (for review see [27]). Therefore, these animals are good candidates for use in xenotransplantation. When five different pig breeds including the Iberian pig were studied in Spain, copy numbers between three and 43 were found and no correlation between the copy number and the heterozygosity or inbreeding coefficient was detected [21]. In another study, approximately 40 copies have been reported in miniature pigs, 30 in Duroc pigs, 20 in Landrace pigs, 25 in Yorkshire pigs and 15 in Korean Jeju pigs analysing DNA from peripheral blood mononuclear cells (PBMCs) [18].

Whereas some studies showed identical numbers of proviruses in different organs of a single pig [19], others have demonstrated differences in the copy number of PERV between different organs of one pig [20,28,29]. This finding was unexpected. On one hand, technical factors such as the way how the DNA has been isolated from different organs, the purity of the DNA, the sensitivity of the PCR, and tissue-specific contaminations affecting the PCR method may contribute to different copy numbers in a single individual. In addition, different cell types can be found in one organ and, more important, a high number of blood cells in the organ may also influence the result. On the other hand, these results may indicate that PERVs were still actively replicating and integrating as new proviruses. In Banna minipigs, differences between 1.9 copies in the lung and 6.3 in the thyroid gland using primers specific for the *gag* gene sequence were reported [20]. This suggests that the lowest number 1.9 indicates the number of the original *gag* endogenous sequences and that in the thyroid gland the number of *gag* sequences increased three times. However, the published number 0 for PERV-A *env* sequences in the pancreas in contrast to 11.2 in the thyroid gland cannot be explained without studying the copy number in the germ line (oocytes, sperm). New investigations using highly purified DNA, sensitive detection methods, excellent standard curves and internal standards are required to study the copy number in different organs of a single pig. Droplet digital™ PCR (ddPCR™) and higher coverage sequencing will be the methods of choice in order to determine the exact copy number of PERV proviruses, however the final determination will be hampered by the identity of multiple sequences of the proviruses.

Regardless of these results, expression of PERV at the RNA and protein level was found to differ significantly from organ to organ (Table 2) [29,30,31,32,33]. Reverse transcriptase PCR (RT-PCR) based methods measuring expression at the mRNA level as well as immunohistochemistry detecting PERV at the protein level have been used to study virus expression. The highest expression was found in the lung, and in the spleen. It is interesting to note that stimulation of pig PBMCs by a mitogen such as the T cell mitogen phytohemagglutinin (PHA) significantly increased virus gene expression. At a certain level of mRNA expression, release of virus particles may be expected [30,34,35,36]. Since the mitogen stimulation simulates an antigenic stimulus of the immune cells, it may also be expected that PERV expression increases when the immune system is stimulated for example due to a bacterial or viral infection. This may explain why the expression in the spleen, being an immune organ, is so high in most cases (Table 2). Based on the reported differences in the copy number [20,28,29], the differences in the expression may be due not only to different transcription levels, but also to the different copy number of proviruses in the organs. However, when the number of proviruses was identical in all organs, significant differences in the level of expression of PERV in different organs were also observed [19].

## 3. PERV Activity and Cellular Restriction Factors

Two main reasons may be discussed to explain the fact that HERVs are inactive in humans and that PERVs are highly active in pigs. First, pigs were infected with the PERV precursor only 7.5 million year ago, whereas the HERV precursors entered the human ancestral primate species around 28 million years. Second, during evolution, primates including humans have developed new and more powerful restriction mechanisms with the goal of inactivating the endogenous viruses more efficiently. APOBEC3 (apolipoprotein B mRNA-editing catalytic polypeptides, A3) proteins belong to these restriction factors; they deaminate DNA cytosines and block the replication of retroviruses and retrotransposons. Each *A3* gene encodes a protein with one or two conserved zinc-coordinating Z motifs. In pigs and in mice, also known to release infectious endogenous retroviruses, there are one A3 protein and two Z motifs, whereas in humans seven proteins and 11 Z motifs were found, indicating that during evolution the protective potency of the host increased [37].

The mechanism on how retroviruses counteract or escape the A3s from their own host species is not well studied [38]. In the case of human immunodeficiency virus type 1 (HIV-1), the amount of A3G in cells infected with wild-type HIV-1 is dramatically reduced by a Vif-dependent degradation process via the ubiquitination-proteasome pathway [39,40,41]. However, little is known about A3-neutralising strategies used by retroviruses that do not encode a Vif protein or the foamy virus (FV) protein Bet [42,43], both inhibiting the incorporation of A3 proteins into the virions. The mechanism of resistance of MuLVs to murine A3 (muA3) is still unclear [44,45,46,47,48]. However, recent data showed that muA3 is an important in vivo restriction factor of the Friend virus complex and the MoloneyMuLV [49,50,51]. Initial studies showed that porcine (po) A3Z2-Z3 strongly inhibits HIV-1 and weakly restricts MuLV [52].

Furthermore, it was reported that overexpressed poA3Z2-Z3 did not significantly interfere with PERV transmission, and it was concluded that PERV was resistant to its species-specific A3 protein [53]. Subsequently, the chromosomal porcine A3 locus for poA3Z2 and poA3Z3 was re-analysed [54,55]. The two A3 genes in pigs encode at least four different mRNAs: A3Z2, A3Z3, A3Z2-Z3, and A3Z2-Z3 splice variant A (SVA). It was found that PERV was significantly inhibited by various porcine A3s in single-round as well as in spreading virus assays. PERV inhibition strongly correlated with a specific cytidine deamination in viral genomes in the trinucleotide 5’TGC for poA3Z2 as well as poA3Z2-Z3 and 5’CAC for A3Z3 [54,55,56]. These results strongly suggest that human and porcine A3s could inhibit PERV replication in vivo, thereby reducing the risk of potential infection of human cells by PERV in the course of pig-to-human xenotransplantation.

In addition to APOBEC other restriction factors were described: the α isoform of tripartite motif-containing protein 5 (TRIM5α), which disrupts the viral capsid (CA) after cell entry, tripartite Motif-Containing Protein 28 (TRIM28), which blocks viral transcription, zinc-finger antiviral protein (ZAP), which directs degradation of viral RNAs, and tetherin, which traps virions on the surface of infected cells [57,58,59,60]. PERV-A and PERV-A/C are insensitive to restriction by TRIM5α molecules in permissive feline Crandall-Reese feline kidney (CRFK) cells expressing TRIM5α proteins from human, African green monkey, rhesus macaque, squirrel monkey, rabbit or cattle [61]. However, overexpression of either human or porcine tetherin in pig cells significantly reduced PERV production [62].

When the capacity of human APOBEC3G (hA3G) and tetherin to prevent PERV infection was compared, hA3G was a stronger inhibitor and a combination of both was the most effective way to restrict PERV [63].

## 4. Evidence for Recombination and de Novo Integration in Vivo

The most convincing evidence for a replication activity of PERVs in vivo is the finding of recombinations between the human tropic PERV-A and the ecotropic PERV-C in pigs. These recombinant PERV-A/C are not integrated in the germ line, but can be found in the cellular DNA from some organs (for review see [27]). They are characterised by a higher replication rate [64,65]. Despite this, first attempts to infect pigs with a PERV-A/C recombinant failed, certainly due to the innate immune system [66].

Recombinations between two defective proviruses may eventually result in a replication competent virus. Such a resurrection of endogenous retroviruses has been observed in antibody-deficient mice [67]. In *Rag1*^−/−^ mice a recombination between the endogenous replication deficient sequence *Emv2* and non-ecotropic virus sequences resulted in viruses able to infect mice. Several recombination events were required to restore *Emv2* infectivity, the resurrected virus infected the germ line and was transmitted as an endogenous retrovirus to progeny. Furthermore, the virus was able to induce tumours. It was shown that the lack of antibodies obviously allowed microbial translocation and stimulation of immune cells by lipopolysaccharides and other bacteria derived mitogens, increasing the expression of endogenous virus sequences and recombination in the stimulated cells. A similar mechanism may be possible in the case of PERV when immune cells were stimulated by microorganisms leading to an enhanced PERV expression and recombination.

Since John Coffin and coworkers found recently unfixed HERV-K sequences in some individuals with intact reading frames and expressed as functional proteins [68], the question arose whether HERVs may recombine with PERVs. Packaging of HERV sequences was undetectable in PERV particles produced from human cells expressing different HERVs [69], indicating that the potential for recombination of PERV and HERV sequences is low and that novel viruses generated by this mechanism are unlikely to represent a risk for xenotransplantation.

## 5. Implications for Pig Testing for Xenotransplantation

To perform a safe xenotransplantation and to prevent PERV transmission to the human recipient, PERV-C free animals with low expression of PERV-A and PERV-B should be selected as donors. To screen animals, easily available source materials such as blood, saliva, ear biopsies, or faeces may be used. If, however, the copy number in the organ needed for transplantation is different when compared with the available source material, additional investigation of the whole animal, or of sisters or brothers have to be performed.

## 6. Inactivation of PERVs by Gene Editing

First attempts to eliminate the multiple proviruses by gene editing were performed by zinc finger nucleases (ZFN) specific for highly conserved sequences in the *pol* region, however cytotoxic effects were observed, possibly due to the high copy number of the proviruses [70]. Recently the inactivation of 62 PERVs in the immortal pig cell line PK-15 by the clustered regularly interspaced short palindromic repeats/CRISPR-associated nuclease (CRISPR/Cas) technology was shown [26], suggesting that the PERV problem may be solved this way. However, it is still unclear whether it will be possible to inactivate all or at least the replication-competent PERVs in the genome and to generate healthy piglets by this strategy [71]. To generate PERV free pigs, primary cells have to be treated with PERV-specific CRISPR/Cas and these treated primary cells should be used for nuclear transfer. Since the number of cell divisions of primary cells is limited (Hayflick limit), cloning and selection of cells with PERVs inactivated by CRISPR/Cas may be difficult.

Interestingly, when HIV-1 infected cells have been treated with a HIV-1 specific CRISPR/Cas, insertions and deletions (indels) were observed impairing the function of the provirus [72,73]. Unexpectedly, some indels led to the emergence of replication competent HIV-1 resistant to CRISPR/Cas. These indels were the result of changing the target DNA sequence. Since PERV is replicating under certain circumstances in some cells of the pig, such a resistance against CRISPR/Cas, although very unlikely, may not be excluded. A combinatorial strategy using CRISPR/Cas targeting different sequences in PERV may prevent this [73]. Infection of the germ line with these viruses and transmission to the progeny is highly unlikely.

## 7. Conclusions

There is clear evidence that PERVs replicate in vivo in the pig, and that this activity results in newly integrated proviruses which were absent from the germ line. This has to be considered when animals with a low copy number and low expression of PERV are selected for xenotransplantation. Improved methods to measure the copy number and the expression level will be required when pre-selecting animals for inactivation of PERVs using the CRISPR/Cas technology and demonstrating the absence of expression.

## Figures and Tables

**Table 1:** Reported porcine endogenous retrovirus (PERV) copy numbers in different pig breeds and cell lines.

Pig Breed, Pig Cell Line	Copy Number	Method	Reference
Landrace × Duroc, Meishan, Pietrain	10–23 (PERV-A), 7–12 (PERV-B) 50 (protease)	Southern blot	Le Tissier et al., [14]; Patience et al., [15]
Landrace × Duroc	32–64	PCR titration	Patience et al. [16]
Chinese miniature pigs ^a^	3.95 ± 0.14 to 95.52 ± 2.20	Real-time PCR	Liu et al., [17]
Six breeds in Korea, Duroc, Landrace, Yorkshire	9 to 50		Lee et al., [18]
Seven organs of four Landrace × Jeju pigs	28.0 ± 2.7	Real-time PCR	Yoon et al., [19]
Chinese Banna minipig inbreed ^b^	1.4 to 98.1 (*pol*) ^b^	Real-time PCR	Zhand et al., [20]
Spanish wild boars and commercial pigs	3 to 43 (*pol)* ^c^	Real-time PCR	Quereda et al., [21]
Pietran	11.7 ^d^/1.6 ^e^	Real-time PCR	Mang et al., [22]
Hampshire	14.0/2.2		
Meishan	12.1/2.8		
Wild boar	3.7/0		
Large white	10.2/0		
Dutch landrace	6.7/2.8		
Westran pigs	19 PERV-A 13 PERV-B	Fluorescence in situ hybridization (FISH)	Lee et al., [23]
Auckland Island pigs	3 to 37	Real-time PCR, PCR-based limited dilution assay	Garkavenko et al., [24]
Duroc pig	20 Gamma ^f^ 4 Beta ^g^	Genome wide sequencing	Groenen et al., [25]
PK15 cells	62	Droplet digital PCR	Yang et al., [26]

^a^ Expression 3.66 ± 0.13 to 43.93 ± 2.5 (real-time RT-PCR); ^b^ Highest copy number detected with primers detecting the polymerase gene (*pol*), highest number in spleen, hearts, ileum; ^c^ Primers detecting the polymerase gene (*pol*) of PERV-A, -B and -C; ^d^ PERV-A and PERV-B using PERV-A/B specific *env* primers; ^e^ PERV-C using PERV-C specific *env* primers; ^f^ Gammaretroviruses; ^g^ Betaretroviruses.

**Table 2:** PERV expression in different tissues of different pig breeds.

Animals	Remarks	Heart	Liver	Spleen	Brain	Lung	Muscle	Kidney	Pancreas	Lymph Node	Small Intestine	Skin	Reference
Hybrid Landrace/Jeju	Korea *pol* primers	+	+	+	-	+	-	+++	nt	nt	nt	nt	Yoon et al., [19]
German landrace ^a^	Multitransgenic ^b^ *gag* primers	+	+	+	+	+++	-	-	-	nt	nt	nt	Dieckhoff et al., [30]
German landrace	Non-transgenic *gag* primers	-	+	+++	nt	++	-	-	-	nt	nt	nt	Dieckhoff et al., [30]
MMS Troll ^c^	*gag* primers	nt	nt	+++	nt	nt	nt	nt	nt	nt	nt	-	Dieckhoff et al., [31]
German landrace	Non-transgenic *env* PERV-A primers	+	+	+++	-	+	+	++	nt	++	++	nt	Mazurek et al., [29]
Yucatan micropig	*gag*, *pol* primer PERV-A	-	+	++	nt	+++	nt	-	-	++	++	nt	Bittmann et al., [32]
Yucatan micropig	PERV-C *env* primer	nt	-	+	nt	+++	nt	nt	nt	+	nt	-	Bittmann et al., [32]
Yucatan micropig	Immunohistochemistry Gag and p15E proteins	++	-	+++	++	nt	-	-	-	-	++	nt	Bittmann et al., [32]
German landrace	*pol* primers	+	++	+++	+	+	+	++	nt	++	nt	nt	Semaan et al., [33]

^a^ two of the animals were hybrids German landrace × minipig; ^b^ TRAIL, CD55, CD59; ^c^ MMS, Munich miniature swine. nt: not tested.
